# Supplementary material for: Preoperative Mutational Analysis of Circulating Tumor Cells (CTCs) and Plasma-cfDNA Provides Complementary Information for Early Prediction of Relapse: A Pilot Study in Early-Stage Non-Small Cell Lung Cancer
Source: Cancers (Basel). 2023 Mar 21;15(6):1877. doi: 10.3390/cancers15061877 (PMC10047138; doi:10.3390/cancers15061877)
Supplement: Supplementary file 1 [file cancers-15-01877-s001.zip › cancers-2269226-supplementary.pdf]

Suppl. Table S1: Adjuvant Therapy of each patient after surgery.

	Adjuvant Therapy			
	Chemotherapy			RadioTherapy
A/A	YES / NO	THERAPY	No of cycles	Yes/ No
1	NO			NO
2	YES	Paclitaxel / Carboplan	4	NO
3	YES	Cisplatin / Navelbine	3	NO
4	NO			NO
5	NO			NO
6	NO			NO
7	NO			NO
8	YES	Carboplatin / Vinorelbine	6	NO
9	NAI	Carboplatin / Navelbine	4	NO
10	YES	Navelbine / Carboplan	4	NO
11	YES	Navelbine / Carboplan	4	NO
12	NAI	Carboplatin / Navelbine	4	NO
13	YES	Cisplatin / Navelbine	4	NO
14	YES	Cisplatin / Navelbine	4	NO
15	NO			NO
16	YES	Navelbine / Carboplatin	4	NO
17	YES	Carboplatin / Etoposide	4	NO
18	YES	Vinorelbine / Carboplatin	4	YES
19	YES	Carboplatin / Vinorelbine	4	NO
20	YES	Cisplatin / Navelbine	4	NO
21	YES	Carboplatin / Navelbine	4	
22	NO			NO
23	YES	Navelbine	4	YES
24	YES	Carboplatin / Navelbine	4	NO
25	YES	Carbo/Alimta	4	NO
26	NO			NO
27	YES	Cisplatin / Navelbine	4	NO
28	NO			YES
29	YES	Cisplatin / Vinorelbine	4	NO
30	NO			NO
31	YES	Cisplatin / Navelbine	4	NO
32	YES	Cisplatin / Navelbine	4	YES
33	YES	Cisplatin / Navelbine	4	NO
34	YES	Carboplatin / Navelbine	4	NO
35	YES	Carboplatin / Navelbine	4	YES
36	NO			NO
37	YES	Carboplatin / Navelbine	4	YES

38	NO			NO
39	NO			NO
40	NO	Carbo/Navelbine	4	NO
41	YES			NO
42	NO			NO
43	NO			NO
44	YES	Carbo/Navelbine	4	NO
45	NO			NO
46	YES	Carbo/Navelbine	4	NO
47	NO			NO
48	NO			NO
49	NO			NO

Suppl. Table S2: Multivariate analysis for RFS and OS for NSCLC patients

**RFS**		
Treatment (radiotherapy vs chemotherapy)	1.472 (0.306-7.086)	P=0.618
Histology (Non-Adeno vs Adeno)	1.156 (0.430-3.110)	P=0.960
Stage (I vs II/IIIA)	1.831 (0.705-4.759)	P=0.215
Size (>5cm vs ≤5cm)	0.884 (0.288-2.714)	P=0.830
Smoking (Yes vs No)	0.294 (0.053-1.624)	P=0.294
Lymph nodes (0 vs <1)	1.691 (0.569-5.024)	P=0.345
At least one mutation in CTC-derived DNA (yes vs no)	1.536 (0.592-3.986)	P=0.378
**At least one mutation in plasma-ct DNA (yes vs no)**	**3.990 (1.312-12.133)**	**P=0.015**
**OS**		
Treatment (radiotherapy vs chemotherapy)	0.996 (0.915-1.084)	P=0.924
Histology (Non-Adeno vs Adeno)	0.712 (0.178-2.856)	P=0.632
Stage (I vs II/IIIA)	4.770 (0.531-42.854)	P=0.115
Size (>5cm vs ≤5cm)	1.704 (0.324-8.962)	P=0.529
Smoking (Yes vs No)	1.620 (0.267-9.810)	P=0.596
Lymph nodes (0 vs <1)	1.912 (0.317-11.545)	P=0.492
